# The relationship between family history of cancer, coping style and psychological distress

**DOI:** 10.12669/pjms.303.4634

**Published:** 2014

**Authors:** Yu Liu, Chunmei Cao

**Affiliations:** 1 Yu Liu, Department of Psychology, Tsinghua University, Beijing 10084, China.; 2 Chunmei Cao, Department of Physical Education, Tsinghua University, Beijing 10084, China.

**Keywords:** Anxiety, Cancer-specific stress, Coping style, Depression, Family history of cancer

## Abstract

***Objective: ***To investigate the relationship between family history of cancer, coping style and psychological distress.

***Methods: ***Total 80 patients with family history of cancer and 72 normal controls were analyzed using self-reporting inventory (SCL-90), coping style scale and impact of event scale-revised (IES-R).

***Results:*** 1. Between the two groups of patients, there were significant differences in anxiety, depression, cancer-specific distress and coping style. 2. Psychological distress (anxiety, depression and cancer-specific distress) had positive correlation with negative coping style and family history. 3. Negative coping style played an intermediary role in the family history and psychological distress.

***Conclusion:*** The negative coping style will predispose to a more stronger psychological distress among the individuals with family history of cancer.

## INTRODUCTION

For the study of the relationship between family history of cancer, coping style and psychological distress, the researchers abroad mainly focus on the healthy women with a family history of breast cancer. It has been reported that the cancer-specific distress among women with a family history of breast cancer was higher than that among women without a family history.^1^^,^^2^ The coping style is an important factor in making individual environmental adaptability and psychological health. Previous study has suggested that positive coping style was associated with good psychological adjustment and negative coping style was related to maladjustment and was harmful to individual psychological health.^3^^,^^4^

The experience of immediate family members with cancer is a life stressor to their relatives, which will trigger different cognitions that whether they will suffer from cancer by heredity in the future, lead to different coping styles and different psychological reactions.^5^^,^^6^ Cao et al investigated healthy women with a family history of breast cancer and reported that 46% of them were concerned that they would suffer from breast cancer in the future and 28% of them said that the concern that they would possibly develop breast cancer influenced their daily life.^7^

The morbidity of breast cancer, lung cancer and gastric cancer is at a relatively high level in our country. The present study employed the healthy individuals with a family history of the three kinds of cancer as subjects. Our aim was to discover the coping styles which are bad for individual stress adjustment and find out more effective coping styles by investigating the relationship between family history of cancer, coping style and psychological distress, which will provide valuable information for psychological health intervention.

## METHODS

***Subjects: ***Subjects were divided into two groups. One group was healthy people with a family history of cancer (breast cancer, lung cancer or gastric cancer). We recruited 82 healthy people accompanying the re-examination of cancer patients. The valid sample was 80: male 46, female 34. The average age was 44.5±11.6 years old. The other group was healthy people without a family history of cancer. We recruited 76 health people without a family history in the clinic. The valid sample was 72: male 38, female 34. The average age was 43.8±10.7 years old. The difference of age, sex, educational status and financial income was not statistically significant. 

***Research tools***

***General investigation forms: ***The information collected include sex, age, educational status, career, financial income, the relationship with cancer patients and the duration of cancer.

***Simplified Coping Style Questionnaire: ***The total number of questions was 20, including 12 positive coping questions and 8 negative coping questions.^8^

***Self-reporting inventory (SCL-90): ***All the two dimension questions of anxiety and depression were extracted and the total number was 23.^9^

***Impact of event scale-revised: ***The scale included intrusion, avoidance, and hyperarousal. The total question number was 22. The present study replaced “case” with “cancer”, and the total score of the three factors means the level of cancer-specific distress. The higher the score the more distressed the patients are.^10^

## RESULTS

***The comparison of the level of psychological distress in the two groups: ***There was significant difference between the two groups in anxiety, depression, intrusion, avoidance and hyperarousal (Table-I).

***The relationship between family history of cancer, coping style and psychological distress: ***There was no significant correlation between positive coping style and the five factors (anxiety, depression, intrusion, avoidance and hyperarousal) mentioned above. The family history and negative coping style had positive correlation with those factors (Table-II).

***The intermediary role of negative coping style in the family history and psychological distress: ***The present study employed the mediating effect testing model of three steps to investigate the intermediary role of negative coping style in the family history and psychological distress (anxiety, depression and cancer-specific distress). First, we regarded family history as the argument and negative coping style as the dependent variable to investigate the predictive effect of family history on negative coping style; second, we regarded negative coping style as the argument and anxiety (depression, cancer-specific distress) as the dependent variable to investigate the predictive effect of negative coping style on anxiety (depression, cancer-specific distress); at last, hierarchical regression analysis was used to investigate the effect of family history on anxiety (depression, cancer-specific distress) when negative copign style was taken into consideration. The results suggested that negative coping style had significant positive predictive effect on anxiety (depression, cancer-specific distress), but the predictive effect of family history decreased when demographic variables were controlled (Table III and IV).

**Table-I:** The comparison of the level of psychological distress in the two groups

*Group*	*Anxiety *	*Depression*	*Intrusion*	*Avoidance*	*H* *yperarousal*
Without family history	1.37±0.36	1.96±0.51	5.83±1.92	8.86±2.19	6.37±1.61
With family history	2.15±0.52**	3.06±0.73**	10.22±2.38**	12.27±3.1*	8.16±2.04*

* P<0.05,

** P<0.01 vs. the group without family history.

**Table-II:** The relationship between family history of cancer, coping style and psychological distress

	*Anxiety*	*Depression*	*Intrusion*	*Avoidance*	*Hyperarousal*
Family history	0.419	0.382	0.461	0.497	0.443
Positive coping style	-0.261	-0.302	-0.182	-0.227	-0.176
Negative coping style	0.486	0.611	0.372	0.594	0.570

**Table-III:** The intermediary effect of negative coping style on anxiety and depression

	*Negative coping style*		*Anxiety (Depression)*		*Anxiety (Depression)*		
	*Step 1*	*Step 2*	*Step 1*	*Step 2*	*Step 1*	*Step 2*	*Step 3*
*Step 1 Demographic variables*							
Sex	-0.108	-0.110	-0.134(-0.169)	-0.121(-0.152)	-0.134(-0.169)	-0.140(-0.176)	-0.131(-0.158)
Age	0.317	0.326	0.127(0.116)	-0.163(-0.194)	0.127(0.116)	0.132(0.127)	-0.137(-0159)
Economic income	0.197	0.195	0.252(0.108)	0.212(-0.137)	0.252(0.108)	0.239(0.100)	0.216(-0.141)
*Step 2*							
Family history		0.374				0.557(0.542)	0.481(0.502)
*Step 3*							
Negative coping style				0.491(0.563)			0.391(0.459)
△F	2.681	8.653	1.593(0.273)	17.22(26.81)	0.821(0.273)	25.49(32.85)	10.95(18.43)
R^2^	0.128	0.241	0.106(0.381)	0.256(0.281)	0.132(-0.124)	0.291(0.304)	0.361(0.418)
△R^2^	0.176	0.183	0.138(0.102)	0.253(0.318)	0.130(0.102)	0.302(0.337)	0.182(0.226)

**Table-IV:** The intermediary effect of negative coping style on cancer-specific distress

	*Negative coping style *		*cancer-specific distress*		*cancer-specific distress*		
	*Step 1*	*Step 2*	*Step 1*	*Step 2*	*Step 1*	*Step 2*	*Step 3*
*Step 1 Demographic variables*							
Sex	-0.108	-0.110	-0.116	-0.101	-0.116	-0.162	-0.128
Age	0.317	0.326	0.359	0.281	0.359	0.114	0.274
Economic income	0.197	0.195	0.161	0.104	0.161	0.146	0.172
*Step 2*							
Family history		0.374				0.407	0.351
*Step 3*							
Negative coping style				0.463			0.431
△F	2.681	8.653	3.372	16.418	3.308	12.328	13.081
R^2^	0.128	0.241	0.141	0.275	0.136	0.232	0.332
△R^2^	0.176	0.183	0.172	0.240	0.172	0.201	0.206

Ps: Cancer-specific distress refers to the sum of intrusion, avoidance and hyperarousal.

## DISCUSSION

With the development of material civilization and spiritual civilization in human society, the demand for life standard and life quality is becoming increasingly high. At the same time, with the alteration of environment and lifestyle, cancer has been one of the most serious diseases threatening people’ s life. Cancer not only brings psychological distress to patients, but also influences patients’ family members dramatically. And the mental health status of family members is directly related to the mood and life quality of patients.^11^

Often, some patients ask the doctors whether people will suffer from cancer by hereditary and whether cancer can be prevented. For those questions, it is hard to draw a conclusion simply. Plenty of medical practices suggest that hereditary factor does play a role in the development of tumor.^12^ Then, how does familial heredity phenomenon of cancer come about? At present, it is possibly due to chromosome aberration.^13^ There are 46 chromosomes in every cell of normal person. Various of carcinogens trigger chromosome aberration, which means the chromosome is different from that in normal cells in terms of number and morphology. The chromosome aberration can sometimes be passed on to offspring, which makes the next generation have the possibility of suffering from cancer.^14^ But having the possibility of suffering from cancer does not mean suffering from cancer, that is to say the chance of suffering from cancer is higher than that of normal people. Generally speaking, cancer is related to hereditary to some extent. For the people with cancer family history, on one hand, they need to realize that although they may suffer from cancer due to hereditary, they should avoid unnecessary fear; on the other hand, they need to pay more attention on the prevention of cancer, and try to detect, diagnose and treat cancer as soon as possible.^15^

About 10%-15% of cancer is due to hereditary.^16^ In the epidemiological investigation, there exists records about familial cancer.^17^ The heritability of colorectal cancer, breast cancer, retinoblastoma and lung cancer was the highest.^18^ Although familial cancer suggests that cancer has heredity, it does not mean that all the people will suffer from cancer by hereditary. Genetic research has found that cancer itself cannot be genetic, and what people inherit is the susceptibility to cancer.^19^ Most cancer is the result of interaction between genetic and environmental factors. The common living environment and lifestyle are intended to trigger the same cancer in a family. At present, some kinds of cancer, such as colorectal cancer and breast cancer, have genetic predisposition.^20^

Different coping styles can affect individual's emotional state, and further affects mental health status. The present study demonstrates that patients with family history appears much stronger anxiety, depression and cancer-specific distress compared with patients without family history. On the other hand, family history has significant predictive effect on psychological distress, which is associated to the cognition that the patients with family history will possibly suffer from cancer in the future. Coping style sometimes plays a regulatory role, sometimes a intermediary role between stress and psychological reaction. The regression analysis in the present study suggests that negative coping style had significant positive predictive effect on anxiety (depression, cancer-specific distress), but the predictive effect of family history decreased when demographic variables were controlled. Part of the intermediary role of negative coping style between family history and psychological distress is verified. The results suggest that the patients will experience higher levels of negative emotions and psychological distress by employing negative coping style to stress. The patients with a family history of cancer will be disturbed by the “hereditary” cognition, and are intended to take negative coping style, which will trigger maladjustment. Consequently, negative emotion appears, especially the cancer-specific distress, which is harmful to mental health.
